# Supplementary material for: Changes and prognostic value of cardiopulmonary exercise testing parameters in elderly patients undergoing cardiac rehabilitation: The EU-CaRE observational study
Source: PLoS One. 2021 Aug 3;16(8):e0255477. doi: 10.1371/journal.pone.0255477 (PMC8330933; doi:10.1371/journal.pone.0255477)
Supplement: S1 Fig — Panel A shows patients after cardiac surgery and no surgery. Panel B shows patients by CPET risk score (reduced peak VO2, VE to VCO2 slope and/or OUES based on the cut-offs derived from this study). VO2, oxygen uptake; VE, ventilation; VCO2, carbon dioxide ouput; OUES, oxygen uptake efficency slope. (DOCX) [file pone.0255477.s001.docx]

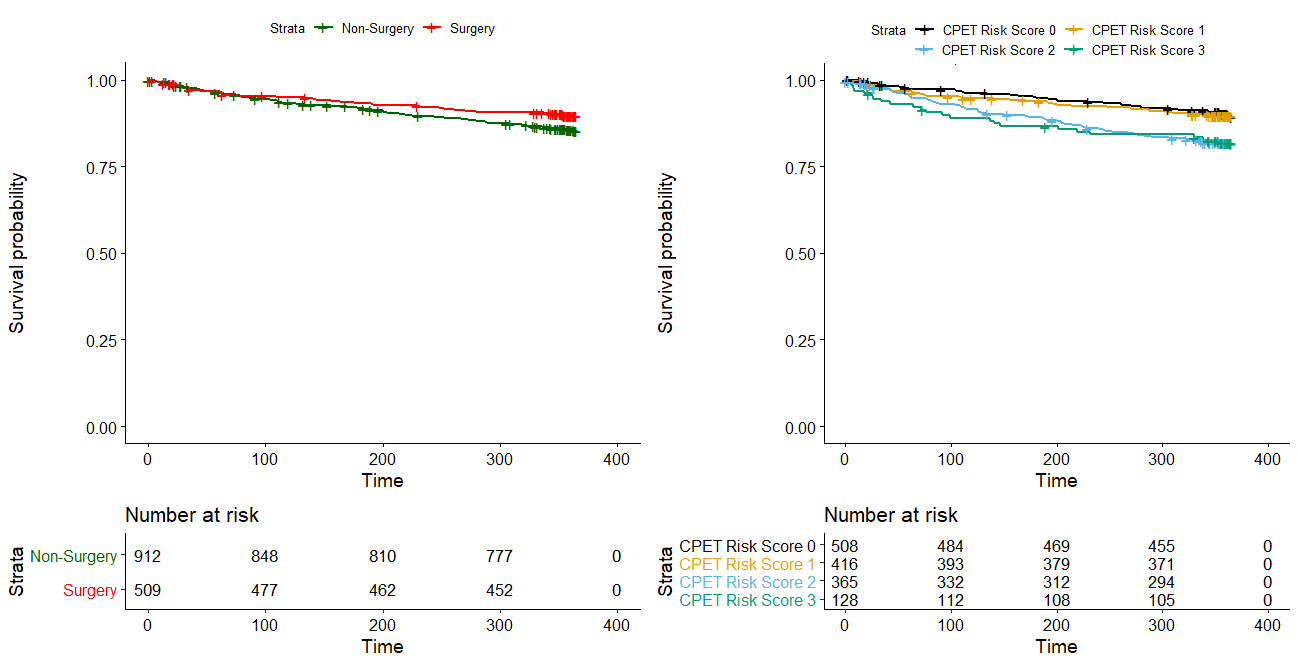


S1 Fig: Kaplan-Meier Curves for major adverse cardiovascular events within 365 days after cardiac rehabilitation entry. Panel A shows patients after cardiac surgery and no surgery. Panel B shows patients by CPET risk score (reduced peak VO2, VE to VCO2 slope and/or OUES based on the cut-offs derived from this study)

VO2, oxygen uptake; VE, ventilation; VCO2, carbon dioxide ouput; OUES, oxygen uptake efficency slope
