# Supplementary material for: Changes and prognostic value of cardiopulmonary exercise testing parameters in elderly patients undergoing cardiac rehabilitation: The EU-CaRE observational study
Source: PLoS One. 2021 Aug 3;16(8):e0255477. doi: 10.1371/journal.pone.0255477 (PMC8330933; doi:10.1371/journal.pone.0255477)
Supplement: S1 Table — (DOCX) [file pone.0255477.s002.docx]

S1 Table: Multiple logistic mixed models for major adverse cardiac events:

Model 1 Model 2 Model 3 Model 4

-----------------------------------------------------------------------------------------------------------------------

(Intercept) -2.21 [-2.88; -1.54] * -2.00 [-2.60; -1.41] * -2.19 [-2.86; -1.53] * -2.12 [-2.75; -1.49] *

Age [SD] vv 0.01 [-0.20; 0.21] 0.02 [-0.18; 0.22] 0.00 [-0.20; 0.21] 0.01 [-0.19; 0.22]

Male Sex -0.00 [-0.51; 0.51] -0.18 [-0.67; 0.30] 0.01 [-0.51; 0.53] -0.10 [-0.59; 0.40]

Time from Index event [SD]0.08 [-0.15; 0.31] 0.10 [-0.12; 0.32] 0.09 [-0.14; 0.31] 0.10 [-0.13; 0.33]

PCI 0.55 [ 0.10; 1.01] * 0.46 [ 0.02; 0.90] * 0.50 [ 0.05; 0.94] * 0.53 [ 0.08; 0.98] *

VO2 [SD -0.31 [-0.56; -0.07] *

VE to VCO2 slope 0.21 [ 0.01; 0.42] *

OUES [SD] -0.29 [-0.53; -0.05] *

VE to VCO2 to VO2 [SD] 0.27 [ 0.07; 0.47] *

-----------------------------------------------------------------------------------------------------------------------

AIC 1125.14 1127.80 1125.33 1122.90

BIC 1161.96 1164.60 1162.13 1159.69

Log Likelihood -555.57 -556.90 -555.66 -554.45

Num. obs. 1421 1417 1418 1417

Num. groups: Centre 8 8 8 8

Var: Centre (Intercept) 0.13 0.08 0.11 0.10

=======================================================================================================================

* Null hypothesis value outside the confidence interval.

Model 5 Model 6 Model 7 Model 8 Model 9

-----------------------------------------------------------------------------------------------------------------------------------------------

(Intercept) -1.98 [-2.76; -1.19] * -2.24 [-2.92; -1.57] * -2.00 [-2.64; -1.35] * -1.99 [-2.64; -1.34] * -2.98 [-3.96; -2.00] *

Age [SD] 0.10 [-0.13; 0.33] 0.01 [-0.20; 0.21] 0.00 [-0.21; 0.22] 0.03 [-0.18; 0.24] -0.01 [-0.22; 0.19]

Male Sex -0.27 [-0.87; 0.32] 0.08 [-0.47; 0.62] -0.23 [-0.73; 0.28] -0.19 [-0.69; 0.32] 0.00 [-0.50; 0.51]

Time from Index event [SD 0.12 [-0.13; 0.38] 0.09 [-0.14; 0.32] 0.11 [-0.12; 0.34] 0.10 [-0.13; 0.33] 0.10 [-0.13; 0.33]

PCI 0.39 [-0.11; 0.89] 0.51 [ 0.05; 0.96] * 0.46 [-0.02; 0.94] 0.43 [-0.04; 0.89] 0.56 [ 0.11; 1.01] *

VT1 -0.10 [-0.36; 0.17]

O2 pulse -0.29 [-0.53; -0.04] *

HR Recovery -0.17 [-0.41; 0.07]

HR reserve -0.11 [-0.35; 0.12]

Cpet risk score 1 0.64 [-0.12; 1.40]

Cpet risk score 2 0.79 [ 0.02; 1.56] *

Cpet risk score 3 1.12 [ 0.35; 1.89] *

-----------------------------------------------------------------------------------------------------------------------------------------------

AIC 885.22 1114.77 1041.52 1069.08 1122.41

BIC 920.43 1151.50 1077.84 1105.55 1169.72

Log Likelihood -435.61 -550.38 -513.76 -527.54 -552.21

Num. obs. 1130 1405 1324 1353 1417

Num. groups: Centre 8 8 8 8 8

Var: Centre (Intercept) 0.21 0.11 0.11 0.12 0.11

===============================================================================================================================================

* Null hypothesis value outside the confidence interval.
