# Supplementary material for: Changes and prognostic value of cardiopulmonary exercise testing parameters in elderly patients undergoing cardiac rehabilitation: The EU-CaRE observational study
Source: PLoS One. 2021 Aug 3;16(8):e0255477. doi: 10.1371/journal.pone.0255477 (PMC8330933; doi:10.1371/journal.pone.0255477)
Supplement: S1 File — (PDF) [file pone.0255477.s003.pdf]

Supplement to Research Plan for submission to Ethics Committee of Switzerland

The (cost) effectiveness, sustainability and participation levels of current EUropean Cardiac Rehabilitation programmes in Elderly: a prospective cohort study

EU-CaRE

Effectiveness of currently available CR programmes from 8 European institutes in elderly patients

Type of Research Project:	Research project in which biological material is sampled from humans and health-related personal data is collected
Risk Categorisation:	Risk category: A (according to HRO Art.7.)
Project Identifier:	NL 52816.075.15
Study Registration:	Nederlands Trial Register<br>NTR5306<br>16. July 2015
Project Leader:	Name: PD Dr. Matthias Wilhelm<br>Address: Universitätsklinik für Kardiologie<br>Inselspital<br>Universitätsspital u. Universität Bern<br>3010 Bern<br>Switzerland<br>Email: Matthias.Wilhelm@insel.ch<br>Phone: +41 (0)31 632-8986

Supplement Version and Date: Version 1.1 of 10-Nov-2015

CONFIDENTIAL
The information contained in this document is confidential and the property of the sponsor. The information may not - in full or in part - be transmitted, reproduced, published, or disclosed to others than the applicable Competent Ethics Committee(s) and Regulatory Authority(ies) without prior written authorisation from the sponsor except to the extent necessary to obtain informed consent from those who will participate in the study.

SIGNATURE PAGE

Project number NL 52816.075.15

Project Title The (cost) effectiveness, sustainability and participation levels of current EUropean Cardiac Rehabilitation programmes in Elderly: a prospective cohort study (EU-CaRE)

The sponsor, the Swiss project leader and the methodologist Dr. P. Koopmans have approved the **research plan (v1.2; 11-Jun-2015)** and the **Supplement for Submission to the Ethics Committee of Switzerland (v1.1; 10-Nov-2015)** and confirm hereby to conduct the study according to the protocol, current version of the World Medical Association Declaration of Helsinki, ICH-GCP guidelines or ISO 14155 norm if applicable and the local legally applicable requirements.

Sponsor:
Dr Arnoud W.J Van't Hof

Place/Date Signature

Swiss Project Leader and Sponsor representative in Switzerland:
PD Dr. Matthias Wilhelm

BERN 18/11/15 M. Wilhelm

Place/Date Signature

Project Methodologist:
Dr. Petra Koopmans

Place/Date Signature

CONFIDENTIAL
The information contained in this document is confidential and the property of the sponsor. The information may not - in full or in part - be transmitted, reproduced, published, or disclosed to others than the applicable Competent Ethics Committee(s) and Regulatory Authority(ies) without prior written authorisation from the sponsor except to the extent necessary to obtain informed consent from those who will participate in the study.

SIGNATURE PAGE

Project number	NL 52816.075.15
Project Title	The (cost) effectiveness, sustainability and participation levels of current EUropean Cardiac Rehabilitation programmes in Elderly: a prospective cohort study (EU-CaRE)

The sponsor, the Swiss project leader and the methodologist Dr. P. Koopmans have approved the **research plan (v1.2; 11-Jun-2015)** and the **Supplement for Submission to the Ethics Committee of Switzerland (v1.1; 10-Nov-2015)** and confirm hereby to conduct the study according to the protocol, current version of the World Medical Association Declaration of Helsinki, ICH-GCP guidelines or ISO 14155 norm if applicable and the local legally applicable requirements.

Sponsor:
Dr Arnoud W.J Van't Hof

Zwolle, 19 nov 2015

Place/Date Signature

Swiss Project Leader and Sponsor representative in Switzerland:
PD Dr. Matthias Wilhelm

Place/Date Signature

Project Methodologist:
Dr. Petra Koopmans

Zwolle, 20 nov 2015 P Koopmans

Place/Date Signature

This document contains additional information to research project protocol NTR5306 "EU-CaRE" for submission to the Swiss ethics committee.

Table of Contents

1. ADMINISTRATIVE STRUCTURE 4

2. ETHICAL AND REGULATORY ASPECTS 5

2.1 Ethical Conduct of Study 5

2.2 Ethics Committee (EC) and Competent Authorities (CA), FOPH 5

2.3 Participant privacy and safety 5

2.4 Study registration 5

3. PROJECT DESIGN 5

3.1 Type of research and general project design 5

3.2 Methods of minimising bias 6

4. PROJECT ASSESSMENTS 6

4.1 Definition of Serious Events (SEs) 6

5. STATISTICAL METHODOLOGY 6

5.1 Data processing 6

5.2 Planned analysis 6

5.2.1 Datasets to be analysed 6

5.2.2 Ancillary analysis 7

5.2.3 Deviations from the original statistical plan 7

6. FUNDING AND SUPPORT 7

7. INSURANCE 7

1. ADMINISTRATIVE STRUCTURE

International Project Leader	Name: Prof. Dr. Eva Prescott<br>Address: Bispebjerg University Hospital<br>Copenhagen (part of Region<br>Hovedstaden)<br>Department of Cardiology<br>Bispebjerg Bakke 23<br>2400 Copenhagen<br>Denmark<br>Email: Eva.irene.bossano.prescott@regionh.dk<br>Phone: +45 (0)2257 2614
Sponsor	Name: Dr. Arnoud W.J Van't Hof<br>Address: Isala<br>Dr Arnoud W.J Van't Hof<br>Dep of cardiology<br>Postbus 10400<br>8000 GK Zwolle<br>The Netherlands<br>Email: a.w.j.vant.hof@isala.nl<br>Phone: +31 (0)38 4244361
Sponsor representative in Switzerland and Swiss Project Leader	Name: PD Dr. Matthias Wilhelm<br>Address: Universitätsklinik für Kardiologie<br>Inselspital<br>Universitätsspital u. Universität Bern<br>3010 Bern<br>Switzerland<br>Email: Matthias.Wilhelm@insel.ch<br>Phone: +41 (0)31 632-8986
Project site(s) and responsible researcher:	Please refer to protocol appendix II and III.
Methodologist / Biostatistician:	Name: Dr. Petra Koopmans<br>Address: Diagram B.V.<br>Dokter Stolteweg 96<br>8025 AZ Zwolle<br>The Netherlands<br>Email: p.koopmans@diagram-zwolle.nl<br>Phone: +31 (0) 384262999<br>Fax: +31 (0) 38 4262990
International CRO	Diagram B.V.<br>Dokter Stolteweg 96<br>8025 AZ Zwolle<br>The Netherlands
CRO for Switzerland	Venn Life Sciences GmbH<br>Marie-Curie-Str. 2<br>53359 Rheinbach<br>Germany

2. ETHICAL AND REGULATORY ASPECTS

2.1 Ethical Conduct of Study

The research project will be carried out in accordance to the research plan and with principles enunciated in the current version of the Declaration of Helsinki (DoH), the Essentials of Good Epidemiological Practice issued by Public Health Schweiz (EGEP), the Swiss Law and Swiss regulatory authority's requirements as applicable. The EC and regulatory authorities will be informed about project start and termination.

2.2 Ethics Committee (EC) and Competent Authorities (CA), FOPH

The regular end, premature end or interruption of the research project is reported to the EC within 90 days upon completion of the project (HRO Art. 22).

2.3 Participant privacy and safety

The Project Leader affirms and upholds the principle of the participants' right to dignity, privacy and health and that the project team shall comply with applicable privacy laws. Especially, anonymity of the participants shall be guaranteed when presenting the data at scientific meetings or publishing them in scientific journals.

Individual participant medical information obtained as a result of this research project is considered confidential and disclosure to third parties is prohibited. Once a patient has disconfirmed his or her data remain anonymised after completion of data analysis.

Participant confidentiality will be further ensured by utilising identification code numbers to correspond to medical information in the computer files. Once a patient is included in the study (and entered in the eCRF) every participant get its own eCRF number.

The eCRF number is generated by the randomization system from a list per study site, normally in sequence. Patients' CRF numbers are unique numbers and a number set per site cannot be mixed up between other sites. The code list (for identification of the patient) is stored at the study centre and only the study staff of the study centre has access to this code list.

In the eCRF only year of birth (not date of birth) and gender is registered.

For data verification purposes, authorised representatives of the Sponsor, a competent authority (e.g. FOPH), or an ethics committee may require direct access to parts of the medical records relevant to the project, including participants' medical history.

2.4 Study registration

The study has been registered in the "Nederlands Trial Register" with number NTR5306 on July 16th 2015.

3. PROJECT DESIGN

3.1 Type of research and general project design

The design used for this study is a prospective cohort study, investigator initiated, multicentre conducted in several countries in Europe.

The trial is monocentric in Switzerland.

3.2 Methods of minimising bias

The modality for the exercise test in order to define physical fitness can be a treadmill or cycle and might differ per centre. However, all tests will be performed by the same protocol to standardise as much as possible. For patients not able to participate in a VO_2 peak test, this will be replaced by a 6 Minutes Walking Test. The modality chosen per centre at T0, will remain the same at T1 and T2.

4. PROJECT ASSESSMENTS

4.1 Definition of Serious Events (SEs)

A **serious event** is any unfavourable event for which a causal relationship to sampling of biological material or the collection of health related personal data cannot be ruled out, and which:

- requires hospitalisation or prolongation of an inpatients' hospitalisation,
- results in persistent or significant disability or incapacity, or
- is life-threatening or results in death,

If a serious event occurs the research project will be set on hold.

5. STATISTICAL METHODOLOGY

5.1 Data processing

If necessary, data will be transformed before the statistical analyses will be performed to make distributions closer to the normal distribution.

The following data will be derived:

VO_2 peak will be registered as the mean of highest 30 sec measurement during the cardiopulmonary exercise test.

MACE will be scored when one of the following events has occurred: cardiovascular mortality, all-cause mortality, near sudden cardiac death, ACS, CV intervention/surgery, CV hospital admission and/or CV emergency visits.

Questionnaire scores (PHQ-9, GAD-7, SF36v2) will be calculated from the individual items according to the coding schemes from their respective manuals. The PHQ-9 and the GAD-7 will yield a total score. From the SF36v2 a Physical Component Summary Score and Mental Component Summary Score will be derived.

Care utilisation will be computed as follows: sum of number of admissions, number of emergency visits and number of cardiac interventions (PCI, CABG) between T1 and T2.

5.2 Planned analysis

5.2.1 Datasets to be analysed

All analyses will be performed on the intention to treat population. The ITT population consists of all patients included in the observational study regardless whether or not they completed the CR programme.

5.2.2 Ancillary analysis

When needed missing values will be imputed by multiple imputation methods and the results will be presented as sensitivity analyses.

5.2.3 Deviations from the original statistical plan

Since this is an observational study deviations from the pre-specified analysis are a possibility. Deviations will be explained and reported. If possible results for the pre-specified analysis will also be presented.

6. FUNDING AND SUPPORT

The European research project is funded by "Schweizerischen Staatsekretariat für Bildung, Forschung und Innovation" SBFI (Contract.-No.: 15.0139) and the Eurpean Commission, DG research & Innovation (Grant agreement No. 634439 - EU-CaRE).
The Swiss site is no beneficiary of the EU funding.
The total amount is for trials EU-CaRE and EU-CaRE RCT respectively.

There is no conflict of interest.

7. INSURANCE

As this research project has been categorised in category A due to only minimal risks and inconveniences to the patient, no insurance is needed in Switzerland.
