# Supplementary material for: Changes and prognostic value of cardiopulmonary exercise testing parameters in elderly patients undergoing cardiac rehabilitation: The EU-CaRE observational study
Source: PLoS One. 2021 Aug 3;16(8):e0255477. doi: 10.1371/journal.pone.0255477 (PMC8330933; doi:10.1371/journal.pone.0255477)
Supplement: Attachment — Submitted filename: Response to Reviewers.docx [file pone.0255477.s005.docx]

Response to Reviewers

*We thank the reviewers for their constructive comments and hope we have addressed them accordingly. Please find below our responses to the reviewers’ comments in red font.*

**Journal Requirements:**

*When submitting your revision, we need you to address these additional requirements.*

*1. Please ensure that your manuscript meets PLOS ONE's style requirements, including those for file naming. The PLOS ONE style templates can be found at*

*https://journals.plos.org/plosone/s/file?id=wjVg/PLOSOne_formatting_sample_main_body.pdf and*

*https://journals.plos.org/plosone/s/file?id=ba62/PLOSOne_formatting_sample_title_authors_affiliations.pdf*

*We have adapted the manuscript style according to the template.*

*2. Thank you for including your ethics statement: "The study was approved by all relevant medical ethics committees of all participating centres, and registered at trialregister.nl (NTR5306)."*

*Please amend your current ethics statement to include the full name of the ethics committee/institutional review board(s) that approved your specific study.*

*Once you have amended this/these statement(s) in the Methods section of the manuscript, please add the same text to the “Ethics Statement” field of the submission form (via “Edit Submission”).*

*For additional information about PLOS ONE ethical requirements for human subjects research, please refer to http://journals.plos.org/plosone/s/submission-guidelines#loc-human-subjects-research.*

*Done*

*3. Thank you for stating the following in the Funding Section of your manuscript:*

*"For the Swiss consortium partner, funding was received by the*

*Swiss State Secretariat for Education, Research and Innovation under contract number 15.0139."*

*We note that you have provided funding information that is not currently declared in your Funding Statement. However, funding information should not appear in the Acknowledgments section or other areas of your manuscript. We will only publish funding information present in the Funding Statement section of the online submission form.*

*Please remove any funding-related text from the manuscript and let us know how you would like to update your Funding Statement. Currently, your Funding Statement reads as follows:*

*"The study was funded by the European Union’s Horizon 2020 research and innovation program under grant agreement number 634439 (Arnoud Van't Hoff) and by the Swiss State Secretariat for Education, Research and Innovation for the Swiss consortium partner (Matthias Wilhelm)."*

*Please include your amended statements within your cover letter; we will change the online submission form on your behalf.*

*We removed the funding section from the manuscript and would like to state the funding as follows:*

*For the Swiss consortium partner (TM, PE, MW), funding was received by the Swiss State Secretariat for Education, Research and Innovation under contract number 15.0139. All other authors received funding by the European Union’s Horizon 2020 research and innovation programme under grant agreement No 634439. The funder provided support in the form of salaries for authors [TM, PE, LP, EK, WB, AvV], but had no role in study design, data collection and analysis, decision to publish, or preparation of the manuscript. None of the commercial partners who provided financial support to any of the coauthors had any role in the perception, conduction, analysis, interpretation nor dissemination of this study.*

*Thank you for changing the online form on our behalf.*

*4. We note that the grant information you provided in the ‘Funding Information’ and ‘Financial Disclosure’ sections do not match.*

*When you resubmit, please ensure that you provide the correct grant numbers for the awards you received for your study in the ‘Funding Information’ section.*

*Done*

*5. Thank you for stating the following in the Competing Interests section:*

*"I have read the journal's policy and the authors of this manuscript have the following competing interests: AWJvH reports grants from Medtronic, grants and personal fees from Astra Zeneca, outside the submitted work, UZ reports grants and personal fees from Astra Zeneca, grants and personal fees from Bayer, personal fees from Boehringer Ingelheim, grants and personal fees from BMS, personal fees from Daiichi Sankyo, personal fees from Eli Lilly, grants and personal fees from Novartis, grants and personal fees from MSD, personal fees from Trommsdorf, personal fees from Amgen, outside the submitted work. The other authors have no conflict of interest to declare."*

*We note that one or more of the authors are employed by a commercial company: Diagram B.V.*

*5.1. Please provide an amended Funding Statement declaring this commercial affiliation, as well as a statement regarding the Role of Funders in your study. If the funding organization did not play a role in the study design, data collection and analysis, decision to publish, or preparation of the manuscript and only provided financial support in the form of authors' salaries and/or research materials, please review your statements relating to the author contributions, and ensure you have specifically and accurately indicated the role(s) that these authors had in your study. You can update author roles in the Author Contributions section of the online submission form.*

Diagram B.V. is a contract research organization, however, the present study was an observational study on effects of clinically indicated CR, so there was neither an intervention nor a treatment involving any commercial interests. Diagram received the salaries for L.P. and E.K. from the EC Horizon2020 grant.

*Please also include the following statement within your amended Funding Statement.*

*“The funder provided support in the form of salaries for authors [insert relevant initials], but did not have any additional role in the study design, data collection and analysis, decision to publish, or preparation of the manuscript. The specific roles of these authors are articulated in the ‘author contributions’ section.”*

*If your commercial affiliation did play a role in your study, please state and explain this role within your updated Funding Statement.*

*None of the commercial affiliations or supports played any role in this study. This is now also stated in the Funding Section, which now reads:*

*“For the Swiss consortium partner (TM, PE, MW), funding was received by the Swiss State Secretariat for Education, Research and Innovation under contract number 15.0139. All other authors received funding by the European Union’s Horizon 2020 research and innovation programme under grant agreement No 634439. The funders provided support in the form of salaries for authors [TM, PE, LP, EK, WB, AvV], but had no role in study design, data collection and analysis, decision to publish, or preparation of the manuscript. None of the commercial partners who provided financial support to any of the coauthors had any role in the perception, conduction, analysis, interpretation nor dissemination of this study.”*

*5.2. Please also provide an updated Competing Interests Statement declaring this commercial affiliation along with any other relevant declarations relating to employment, consultancy, patents, products in development, or marketed products, etc.*

*Within your Competing Interests Statement, please confirm that this commercial affiliation does not alter your adherence to all PLOS ONE policies on sharing data and materials by including the following statement: "This does not alter our adherence to PLOS ONE policies on sharing data and materials.” (as detailed online in our guide for authors http://journals.plos.org/plosone/s/competing-interests) . If this adherence statement is not accurate and there are restrictions on sharing of data and/or materials, please state these. Please note that we cannot proceed with consideration of your article until this information has been declared.*

*Please include both an updated Funding Statement and Competing Interests Statement in your cover letter. We will change the online submission form on your behalf.*

*Please know it is PLOS ONE policy for corresponding authors to declare, on behalf of all authors, all potential competing interests for the purposes of transparency. PLOS defines a competing interest as anything that interferes with, or could reasonably be perceived as interfering with, the full and objective presentation, peer review, editorial decision-making, or publication of research or non-research articles submitted to one of the journals. Competing interests can be financial or non-financial, professional, or personal. Competing interests can arise in relationship to an organization or another person. Please follow this link to our website for more details on competing interests: http://journals.plos.org/plosone/s/competing-interests*

*We have included the following Conflict of Interest and Data sharing statements in our cover letter:*

*AWJVH reports grants from Medtronic, grants and personal fees from Astra Zeneca, outside the submitted work, UZ reports grants and personal fees from Astra Zeneca, grants and personal fees from Bayer, per-sonal fees from Boehringer Ingelheim, grants and personal fees from BMS, personal fees from Daiichi Sankyo, personal fees from Eli Lilly, grants and personal fees from Novartis, grants and personal fees from MSD, personal fees from Trommsdorf, personal fees from Amgen, outside the submitted work. LP and EK work for Diagram B.V., a contract research organization. These commercial affiliations do not alter our adherence to all PLOS ONE policies on sharing data and materials. All other authors have no Conflict of Interest to declare.”*

*6. We note that you have indicated that data from this study are available upon request. PLOS only allows data to be available upon request if there are legal or ethical restrictions on sharing data publicly. For information on unacceptable data access restrictions, please see http://journals.plos.org/plosone/s/data-availability*

*In your revised cover letter, please address the following prompts:*

*a) If there are ethical or legal restrictions on sharing a de-identified data set, please explain them in detail (e.g., data contain potentially identifying or sensitive patient information) and who has imposed them (e.g., an ethics committee). Please also provide contact information for a data access committee, ethics committee, or other institutional body to which data requests may be sent.*

*b) If there are no restrictions, please upload the minimal anonymized data set necessary to replicate your study findings as either Supporting Information files or to a stable, public repository and provide us with the relevant URLs, DOIs, or accession numbers. Please see http://www.bmj.com/content/340/bmj.c181.long for guidelines on how to de-identify and prepare clinical data for publication. For a list of acceptable repositories, please see http://journals.plos.org/plosone/s/data-availability#loc-recommended-repositories.*

*There are restrictions to data sharing but these are based on the various legislative restrictions for data containing potentially identifying or sensitive patient information of the involved eight countries and the lack of informed consent to international data sharing. Requests for de-identified aggregated data may be sent to info@diagram-zwolle.nl, Diagram B.V., contract research organization, Dokter Stolteweg 96, 8025 AZ Zwolle, The Netherlands, on behalf of Prof. Arnoud Van’t Hoff, Department of Cardiology, Maastricht University Medical Center, and Cardiovascular Research Institute Maastricht (CARIM), the Netherlands.will update your Data Availability statement on your behalf to reflect the information you provide.*

*7. Please include captions for your Supporting Information files at the end of your manuscript, and update any in-text citations to match accordingly. Please see our Supporting Information guidelines for more information:* [*http://journals.plos.org/plosone/s/supporting-information*](http://journals.plos.org/plosone/s/supporting-information)*.*

*Captions are included at the end of the manuscript, p.18*

**Reviewer Comments:**

*Reviewer #1: PONE-D-20-37659: statistical review

SUMMARY. This is a longitudinal study that (1) tests whether parameters of cardiopulmonary exercise testing (CPET) are significant predictors of major cardiovascular adverse events (MACE) in elderly patients who commence cardiac rehabilitation and (2) seeks factors that explain the greater peak VO2 improvement in surgical compared non-surgical patients. Overall, the statistical analysis seems appropriate, but the authors should describe additional details about the exploited methods (see major issues 1 and 2 below). Furthermore, model diagnostics should be provided (major issue 3).

MAJOR ISSUES

1. The statistical analysis that addresses aim (1) appropriately relies on a mixed effects logistic model. However, Table 2 does not display the parameters of the random effect distribution. I was therefore unable to understand the specific random effect correlation structure that has been chosen for this analysis. The authors should add details about this correlation structure and display the estimated parameters in Table 2.*

Thank you for your comment. We changed the description of in the statistical analysis paragraph on p. 9 to: *Mixed logistic regression models (lme4 package) adjusted for age, sex, PCI, time between index event and start of CR as fixed factors, centre as random intercept and baseline CPET parameters added individually to the model were performed to determine the associations of CPET characteristics with MACE.*

We used the glmer function of the lme4 package in R and did not specify a specific correlation structure.

Table 2 shows the effects for of the CPET parameter of each model, while the other parameters such as the center as random intercepts were not of interest and only used for adjustment. Showing the random effects for each model would make the table very extensive. However, we aimed to show the full model output in the supplementary material as indicated on p. 13:

*From the potentially confounding factors included in the model, only PCI as indication for CR was associated with MACE (Odds ratio ≈ 1.7) while age, sex or time lag of CR uptake did not significantly predict cardiac events (full output shown in supplements).*

Unfortunately, we did not upload the supplements in the first submission, but added them now with the revision. Please see Supporting Information S1 table. The adjusted icc for the models ranged from 0.023 to 0.061, and hence, are quite low.

*2. The analysis of the factors that explain VO2 improvements is based on "robust linear models". However, it is not clear what kind of model has been used by the authors. After all, robustness has many aspects! Robustness with respect to outliers? Robustness with respect to variance assumptions? This should be clarified.*

Thank you for pointing this out. We adapted the statistical analysis paragraph on p9:

*Additionally, linear models robust for outliers (package robustbase) were performed to explore…

3. It seems (last paragraph of the statistical analysis) that traditional model diagnostics have been performed, which is a good idea. However, these results are not displayed nor commented. At least, the results of the diagnostics should be summarized in a supplementary file.*

Thank you for the comment. We added following sentence to the results on p.13

*Analysis of deviance did not indicate a lack of fit in any of the performed logistic models.*

and one sentence on p.14:

*Model diagnostic did not indicate violation of model assumptions in any of the performed robust linear model.*

*Reviewer #2: The study had two objectives:

1) to test the applicability of the previously suggested prognostic value of CPET to elderly cardiac rehabilitation (CR) patients.

2) to explore the underlying mechanism of the greater improvement in exercise capacity (peak oxygen consumption, VO2) after CR in surgical compared to
non-surgical cardiac patients.

Cardiopulmonary exercise tests (CPET) were performed at the start of CR, end of CR, and 1-year follow-up.

Patients were divided into two groups.
- Surgery group: after coronary artery bypass grafting, surgical valve replacement.
- Non-surgery group: percutaneous coronary intervention, percutaneous valve replacement, or without revascularization.

Adverse Cardiac Event (MACE) was defined as a composite endpoint of all-cause and cardiovascular mortality, acute coronary syndrome, aborted sudden cardiac death, cardiovascular intervention/surgery, hospital admission, or emergency visits between T0 and T2.

Results
No CPET parameter further improved the receiver operation characteristics significantly with the model, including only clinical parameters. The higher improvement in peak VO2 (25% vs. 7%) in the surgical group disappeared when adjusted for peak tidal volume and hemoglobin changes.

Conclusion
CPET did not improve the prediction of MACE in elderly CR patients. The more significant improvement of exercise capacity in surgery patients was mainly driven by the restoration of hemoglobin levels and improvement in respiratory function after sternotomy.


My considerations about the article:

It is an interesting paper about cardiovascular rehabilitation (CR) on elderly patients (age 73±5,4, 81% male), with a good number of participants (1,421 for prognostic evaluation and 1,178 for modification on CPET) and multicentric (eight centers in seven countries).


1) Although the CPET aimed a duration of 8-12 minutes, exclusion criteria excluded a protocol duration shorter than 3 minutes. Were CPET with a duration of 3 to 8 minutes included for analyses? These CPETs with short duration (mainly those with less than 5-6 minutes) may have limited the measured variables' utilization and, therefore, the prognostic value.
CPET duration (mean and SD) was not shown in Figure 2 or described in the article.*

Thank you for your comment. CPETs with less than 5-6 minutes were included because of the many fragile patients that did not reach a ramp duration of 5 min. Excluding these patients would have led to a bias. However, we added the following sentences to our limitation section on p.18

*Not all included patients performed a high quality CPET, and in a considerably large proportion (19%) of patients the VT1 could not be determined.* *However, CPET duration was on average 7.75 min (SD 2.7) at baseline and 9.0 min (SD 2.8) at 1-year follow up and therefore of acceptable test duration.*

*2) Existing cut-off values (peak VO2 <18 ml/kg/min, OUES <1550, VE/CO2 slope >31.5) were used to compare the risk of MACE between patients with and without impaired CPET characteristics at the start of CR. [Ref. 16]

[16] Coeckelberghs E, Buys R, Goetschalckx K, Cornelissen VA, Vanhees L. Prognostic value of the oxygen uptake efficiency slope and other exercise variables in patients with coronary artery disease. European journal of preventive cardiology 2016;23(3):237–44.

These cut-offs values were determined by a previous study with a younger population (60,7±9,9 years), all patients with CAD, and a higher peak VO2 (19,5±5,6 ml/kg/min), compared to the surgery group of the presented manuscript (15,3±4,0 ml/kg/min). Peak RER were also higher than surgery or non-surgery group (1,20±0,11 versus 1,07±0,13 or 1,08±0,11).
It is expected that these cut-off values would not apply to the present study due to the study population's characteristic differences and a higher proportion of submaximal CPET.
As shown in Table 2, more than half of the patients were below the cut-off values, especially the peak VO2 values.
peak VO2 <18ml/kg/min 75,9% of patients
OUES <1550 57,4% of patients
VE/CO2 slope >31.5 63,1% of patients

This limitation is described in the Discussion: present study were older and most likely weaker than in the study from Coeckelberghs et al.[16] Hence, the cut-offs were probably not appropriate and the predictive value correspondingly underestimated.

So, the present article should focus on new cut-off values and not the previous. Prognostic cut-off values are always linked to population characteristics.
.
The cut-offs with 95% CI derived from the study population for the non-surgery and surgery group were as follow:
peak VO2, 15.7 [11.8 – 18.1] ml/kg/min and 12.5 [9.8 – 15.7];
OUES, 1.75 [1.2 – 2.1] and 1.35 [0.58 –2.26];
VE/CO2-slope, 50.1 [27.4 – 58.6] and 34.2 [31.5 – 38.2].

The cut-off values were different from the previous study and between the study groups (surgery or non-surgery).”

“The 95% CI of our own cut-offs were wide and may therefore not be applicable for other cohorts.”

Agree. A limited submaximal CPET might have influenced it at T0 for some patients.*

Thank you for your comment. We agree with the reviewer. However, we feel that we have addressed this discrepancy by presenting own cut-offs which, of course, have to be validated in an independent study population and by suggesting which underlying factors could have caused the difference between our study population and the study population from Coeckelsbergh et al. *3) On the abstract, there is information that CPET was performed at the start of CR (T0), end of CR (T1), and 1-year follow-up (T2), but analyses included only T0 and T2 time points.
No results were available for T1. Why?
Analysis should be performed within the 3 CPET groups (T0, T1, and T2).
When treating patients with more severe disease or after procedures, an initial submaximal CPET is expected, so a second CPET (first maximal) is necessary to evaluate these patients better.

Carvalho T, Milani M, Ferraz AS, Silveira AD, Herdy AH, Hossri CAC, et al. Brazilian Cardiovascular Rehabilitation Guideline – 2020. Arq Bras Cardiol. 2020;114(5):943-987.https://doi.org/10.36660/abc.20200407.

Therefore, logistic regression models should include T1 results, besides or instead of T0 results.*

Thank you for this very justified comment. We omitted T1 from analyses due to several reasons:

1) The timing of the second visit (end of rehabilitation) was very different between the participating centers due do different length in cardiac rehabilitation, while T2 was set at 12 month after study inclusion for all centres regardless of duration of rehabilitation.

2) T0 was preferred over T1 because the prognostic value of CPET is, in our opinion, from a clinical perspective higher at the time when patients enter cardiac rehabilitation than when they conclude it.

3) Based on the findings published in *Prescott E, Eser P, Mikkelsen N, Holdgaard A, Marcin T, Wilhelm M, Gil CP, González-Juanatey JR, Moatemri F, Iliou MC, Schneider S, Schromm E, Zeymer U, Meindersma EP, Crocamo A, Ardissino D, Kolkman EK, Prins LF, van der Velde AE, Van't Hof AW, de Kluiver EP. Cardiac rehabilitation of elderly patients in eight rehabilitation units in western Europe: Outcome data from the EU-CaRE multi-centre observational study. Eur J Prev Cardiol. 2020 Nov;27(16):1716-1729. doi: 10.1177/2047487320903869. Epub 2020 Feb 26. PMID: 32102550,* for our second research question (explaining difference between surgery and non-surgery), we expected greater differences between these groups at the start rather than at the conclusion of rehabilitation.*.* Therefore, we considered T0 to T2 to be the most standardized time period with the greatest expected differences between the surgery and non-surgery patient groups.

4) We feel that providing CPET data for all three time points would overload the manuscript.

We agree with the reviewer, however, that a second (maximal) CPET would be optimal. However, this was neither feasible within our study protocol nor would it have likely contributed to a greater number of maximal CPETs given the fragility of many of the included elderly patients. Based on your comment, we performed a post-hoc analysis and added RER >1.05 to the logistic models. However, the inclusion of RER did not alter the effect of peak VO2 on risk of mace. We added the following sentence to our limitation section:

*In addition, the effect of peak VO2 on MACE was not altered when the logistic model was adjusted for RER.*

Other parameter such as OUES or VE/CO2 slope are submaximal parameters and were therefore not influenced by presence or absence of maximal performance.  *4) Table 1 presents an age average of 72.5 (5.3), while the abstract is 73±5.4. Could you please explain its difference?*

The abstract gives the age for the 1421 patients with baseline CPET. Table 1 gives the baseline characteristics for the 1178 patients who have also CPET data at one year follow up.
 *5) Table 1 has only the baseline characteristics of the overall group. It should also include group characteristics (and possible differences) among surgery and non-surgery groups.
It would be even better if patients’ characteristics were described for each procedure: PCI, No revascularization; Percutaneous valve replacement, surgical valve replacement, and CABG.
Possible doubts and bias:
- Had the surgery group patients more diabetes or lower ejection fraction?
- Had PCI patients more acute coronary syndrome?
- Were percutaneous valve replacement patients older?
- Had percutaneous valve replacement patients more COPD? Or were patients older?
-What exactly is a "No revascularization patient?
Stable CAD? Heart failure? Acute coronary syndrome patient not suitable for revascularization? This is not clear in the article.*

Thank you for your comment. We added the information for surgery and non-surgery in Table 1 and renamed “no revascularization” to “Chronic CAD without revascularization”

*6) In the article:
“ Mixed logistic regression models adjusted for age, sex, PCI, time between index event and the start of CR as fixed, centre as random factor and baseline CPET parameters added individually to the model were performed to determine the associations of CPET characteristics with MACE.”

Why was the only PCI included in the regression model?
Why were not other procedures included?
Why was acute coronary syndrome not included?

As described in Table 1: PCI 653 (55%); No revascularization 78 (7%); Percutaneous valve replacement 101 (2%); Surgical valve replacement 79 (7%); CABG 344 (29%).
The manuscript states that "PCI as an indication for CR was associated with MACE (Odds ratio ≈ 1.7)”.
This is because of PCI, or those patients were mainly the ones with the acute coronary syndrome and, consequently, had a higher probability of short-term MACE? This can be a critical bias of the analysis, and this information needs to be more precise.

Possibly there is a requirement to modify and reanalyze the logistic regression models.*

Thank you your comment. Indeed, patients with PCI mostly had an acute coronary syndrome. However, the fact that PCI patients had a higher risk of MACE was a side finding and not part of our research questions. We were interested in the effect of the CPET paramters on risk of MACE and PCI was just used to adjust for group differences. Based on the reviewers comment, we performed a post-hoc analysis and added ACS into the model. However, the effect of peak VO2 on risk of MACE did not change: ACS was not significant, while PCI remained as significant predictor in the model. We therefore suggest to present the logistic models as they are.

Nevertheless, we added the following sentence to the result section on page 13:

*From the potentially confounding factors included in the model, only PCI as indication for CR was associated with MACE (Odds ratio ≈ 1.7), probably driven by the great proportion of patients with PCI after an acute coronary syndrome. Age, sex or time lag of between event and CR uptake did not significantly predict cardiac events. (full output shown in Supporting Information S1 Tables).

7) What was the period between the index event and the CR start?
This data was not described in the article.
CPET completed at a short period after the surgical intervention has limited clinical use and, even more, limited prognostic utility. There can be a risk of complications (bleeding, infection) related to the procedure and not related to baseline diseases.
Esternal pain, exercise discomfort, or even fear to exercise can lead to a submaximal evaluation. The mean peak RER was 1,07 at T0 on the surgery group. So, more than half of the initial CPET were submaximal.
Also, as shown by the actual and previous article, lower hemoglobin levels and limited ventilation response can impact peak VO2 at initial CPET (T0).

That is why I missed the information of CPET at T1, after the end of CR.
A CPET performed 6 to 8 or 12 weeks after the event or procedure could better predict prognosis than an initial limited submaximal CPET in some patients, as previously discussed.*

Thank you for your comment. A previously published article provides the time beween index event and CR start in detail:

*Prescott E, Eser P, Mikkelsen N, Holdgaard A, Marcin T, Wilhelm M, Gil CP, González-Juanatey JR, Moatemri F, Iliou MC, Schneider S, Schromm E, Zeymer U, Meindersma EP, Crocamo A, Ardissino D, Kolkman EK, Prins LF, van der Velde AE, Van't Hof AW, de Kluiver EP. Cardiac rehabilitation of elderly patients in eight rehabilitation units in western Europe: Outcome data from the EU-CaRE multi-centre observational study. Eur J Prev Cardiol. 2020 Nov;27(16):1716-1729. doi: 10.1177/2047487320903869. Epub 2020 Feb 26. PMID: 32102550.*

We agree that maximal CPET is often not possible in fragile elderly patients, especially after surgery. Regarding the comment on submaximal CPET parameters evaluation, we evaluated also the prognostic effects of parameters not requiring maximal testing on MACE. Peak VO2, on the other hand, was not affected when we adjusted for RER as described above.  *8) What was the duration of CR? There is no information about it.
CR duration affected prognosis. Was it evaluated?*

This is a very good point. The cardiac rehabilitation duration differed widely across the centers from two weeks to three months. We have described this in detail in a previous publication:

(*Prescott E, Eser P, Mikkelsen N, Holdgaard A, Marcin T, Wilhelm M, Gil CP, González-Juanatey JR, Moatemri F, Iliou MC, Schneider S, Schromm E, Zeymer U, Meindersma EP, Crocamo A, Ardissino D, Kolkman EK, Prins LF, van der Velde AE, Van't Hof AW, de Kluiver EP. Cardiac rehabilitation of elderly patients in eight rehabilitation units in western Europe: Outcome data from the EU-CaRE multi-centre observational study. Eur J Prev Cardiol. 2020 Nov;27(16):1716-1729. doi: 10.1177/2047487320903869. Epub 2020 Feb 26. PMID: 32102550.)*

By including center as random variable in the model, we have adjusted simultaneously for the duration of CR and other center specific factors such as nationality and socioeconomic factors. However, we aimed to assess the effect of CPET characteristics and not cardiac rehabilitation or patient related characteristics on MACE.

*9) MACE reported: 195 patients (14%) within a mean (SD) follow-up time of 340 (112) days.
14 (1%) allcause-mortality,
11 (1%) CV-mortality
1 (0%) aborted sudden cardiac death.
26 (2%) acute coronary syndromes
121 (9%) CV hospitalizations
107 (8%) CV emergency visits
123 (9%) CV interventions.

I missed a visual graphic of MACE versus time on both study groups. (Kaplan-Meier).

Follow-up was short, as written in Discussion. “In our study, the follow-up period may have been too short, and the definition of MACE too wide to obtain a valuable prediction of MACE.”
Agree. Maybe longer follow-up can provide better results.
Hospitalizations, emergency visits, and interventions could be higher in short-term follow-ups of surgical patients and after acute coronary syndrome.
There is a need for more detailed information about MACE reported.*

Thank you for your suggestions. We agree that hospitalization and emergency visits may occur more often in patients after an ACS. However, we aimed to assess the effect of CPET characteristics on risk of MACE, independently of patient characteristics. Therefore, we adjusted for PCI and some other parameters that we considered to be potentially confounding. As described above, adding ACS to the model did not change the results. Hence, we believe that including PCI as confounding factor in our model sufficiently adjusts for the confounding effect of the most important patient characteristics. Including more patient or CR related characteristics would most likely not change our conclusion, namely that CPET characteristics do not provide much additional prognostic value over patient characteristics.

Unfortunately, a longer follow-up perdiod was not feasible and the study has been concluded in 2019. However, we believe that we have addressed this limitation accordingly in the limitation section. Based on the reviewers comment, we have added Kaplan-Meier curves for non-surgery vs. surgery and for the CPET risk score based on our own cut-offs to the Supporting Information and have added the following sentences to the result section on p13:

*Figure S1 in the supplemental information shows the survival curves for MACE in patients after surgery and non-surgery and patients with the CPET risk score 1-4 based on our own cut-offs, illustrating the distribution of MACE over time.


10) “Mean improvement in peak VO2 was 0.25 l/min higher in surgery patients compared to non-surgery patients. However, the difference declined when adjusting for ΔHb, ΔVT1, or ΔHR reserve was more than halved when adjusted for Δpeak TV and disappeared almost completely when adjusted for ΔHb
and Δpeak TV variables (Figure 3).”*

*Was the Δpeak RER adjust performed?
Peak RER was different between T0 e T2.
A submaximal CPET at T0 might have been compared with a maximal CPET at T2 in the same cases. This could have influenced differences in peak VO2.*

Thank you for your comment. Indeed, the submaximal tests especially in surgery patients may have influenced the results. Based on your comment, we added change in RER to the model. However, the effect sizes did not change substantially when comparing the figure below with Figure 3 included in the manuscript.

*Group Differences adjusted for change in RER*

We therefore suggest to leave Figure 3 as it is. However, we included the following sentence in the result section on p.14:

*Adding change in RER to the model in order to account for the potential confounding effect of submaximal CPETs did not influence the results.


15) Using our own cut-offs did not significantly improve the prediction of MACE (AUC = 66.99, specificity = 52.86, sensitivity = 73.84) compared to the established cut-offs (Table 4)

Table 4 was not available in the document for Review.*

We apologize, it should reference to Table 2. There is no Table 4. We changed it accordingly in the manuscript. *Conclusions:
It is promising but needs to be clearly described and provide more detailed information, as described previously.
Maybe it is necessary to change the logistic regression models.
A longer follow-up may be necessary.
The definition of MACE was too broad and may include short term procedure complications.
Focus on new cut-off values may be better than using previous values derived from a different population.*

Thank you for summarizing your most important concerns regarding our paper. As described above, we believe that the logistic regression models are appropriate. However, we hope to have addressed the reviewer’s concerns by adding additional sentences in the limitation section. Unfortunately, longer follow-up time was not possible, but we hope to have addressed this limitation appropriately.

Indeed, the definition of MACE may include short-term procedure complications. Nevertheless, it seems difficult to distinguish these from other events with the available data and, more importantly, it may also be of interest to detect patients at risk for short term procedure complications. Also, the now included Kaplan-Meier Curves do not indicate a larger proportion of events in the earliest phase of the study.

Regarding cut-offs, we agree with the reviewer. However, we believe that we have addressed this limitation appropriate and we have also provided our new cut-offs which should be validated in a new independent cohort.

*6. PLOS authors have the option to publish the peer review history of their article (*[*what does this mean?*](https://journals.plos.org/plosone/s/editorial-and-peer-review-process#loc-peer-review-history)*). If published, this will include your full peer review and any attached files.


If you choose “no”, your identity will remain anonymous but your review may still be made public.


Do you want your identity to be public for this peer review? For information about this choice, including consent withdrawal, please see our* [*Privacy Policy*](https://www.plos.org/privacy-policy)*.*

*Reviewer #1: No*

*Reviewer #2: No*

*[NOTE: If reviewer comments were submitted as an attachment file, they will be attached to this email and accessible via the submission site. Please log into your account, locate the manuscript record, and check for the action link "View Attachments". If this link does not appear, there are no attachment files.]

While revising your submission, please upload your figure files to the Preflight Analysis and Conversion Engine (PACE) digital diagnostic tool,* [*https://pacev2.apexcovantage.com/*](https://pacev2.apexcovantage.com/)*. PACE helps ensure that figures meet PLOS requirements. To use PACE, you must first register as a user. Registration is free. Then, login and navigate to the UPLOAD tab, where you will find detailed instructions on how to use the tool. If you encounter any issues or have any questions when using PACE, please email PLOS at* [*figures@plos.org*](mailto:figures@plos.org)*. Please note that Supporting Information files do not need this step.*
